# Biodegradation Study of Biomaterials Composed of Acrylated Epoxidized Soybean Oil: An In Vitro Study

**DOI:** 10.1155/bmri/7100988

**Published:** 2024-11-22

**Authors:** Diego Sibilia, Martina Amendolea, Roberta Sangiovanni, Mario Bragaglia, Fabrizio Nicoletti, Pierfrancesco Filetici, Antonio D'Addona, Francesca Nanni, Leonardo Dassatti, Giuseppina Nocca

**Affiliations:** ^1^Dipartimento di Scienze della Vita e Sanità Pubblica, Università Cattolica del Sacro Cuore, Largo F. Vito, 1 00168, Rome, Italy; ^2^Head and Neck Department, School of Dentistry, Fondazione Policlinico Universitario A. Gemelli-IRCCS, Università Cattolica del Sacro Cuore, Largo A. Gemelli 8, Rome 00168, Italy; ^3^Department of Enterprise Engineering, University of Rome “Tor Vergata”, Via del Politecnico 1 00133, Rome, Italy; ^4^Fondazione Policlinico Universitario A. Gemelli, IRCCS, Largo A. Gemelli 8 00168, Rome, Italy; ^5^Dipartimento di Scienze Biotecnologiche di Base, Cliniche Intensivologiche e Perioperatorie, Sezione di Biochimica, Università Cattolica del Sacro Cuore, Largo F. Vito, 1 00168, Rome, Italy

**Keywords:** acrylated oils, biodegradation, cytotoxicity, photopolymerization, regenerative medicine

## Abstract

In the field of regenerative medicine, acrylated epoxidized vegetable oils are emerging as a promising avenue of exploration. The aim of this study is to evaluate the degradability of two formulations of acrylated epoxidized soybean oil (AESO): pure AESO and AESO diluted with soybean oil (SO) for potential bioprintability applications. The comprehensive investigation of these two polymeric formulations included optimization of polymerization conditions, confirmation of cytocompatibility, and, most importantly, the study of their degradability. The results reveal that AESO, used as a biomaterial for biomedical applications, undergoes a distinctive degradation process, combining both enzymatic and oxidative degradation (AESO/SO samples lose 29.45% of their weight after 60 days). This phenomenon is the result of a complex interplay of factors, including the chemical composition and physical characteristics of the polymer, the unique tissue environment in which it is implanted, and the duration of implantation.

## 1. Introduction

Soybean oil (SO) and rapeseed oil, as other vegetable oils, are emerging as promising materials in tissue engineering due to their biodegradability and support for cell growth and differentiation. These oils contain bioactive compounds that positively influence cellular behavior and tissue regeneration [1]. Used in scaffold materials or hydrogels, they provide mechanical support and enhance cell adhesion. Additionally, they can serve as carriers for growth factors, facilitating controlled release within tissue constructs and promoting desired cellular responses [2–6].

Vegetable oils, derived from renewable sources, offer tunable mechanical traits, enhancing their appeal in biomedical applications, among others. Indeed, acrylated epoxidized soybean oil (AESO) is notable as a photo–cross-linkable resin in stereolithography (SLA) and digital light processing (DLP) [6–11]. Preparing AESO for SLA involves acrylating epoxidized SO and adding a photoinitiator for the UV light–induced photopolymerization, enabling the crafting of intricate three-dimensional structures [12]. AESO can also be used in casting, spin coating, and electrospinning to create films, membranes, or porous scaffolds [3]. In terms of applications, AESO-based scaffolds have shown promise in bone tissue engineering by supporting osteogenic differentiation and new bone formation [10, 13]. Moreover, AESO has also been applied as organic polymer matrix to produce antimicrobial biomaterials [14, 15].

In surgical restorative healthcare, a shift toward degradable implant materials is taking place as they could provide temporary support during healing and eventually dissolve, allowing the patient's tissue to replace them [16–18]. Biodegradable polymers, including AESO, are promising candidates for implants due to their ability to undergo hydrolytic, enzymatic, and oxidative degradation [7, 19, 20]. Indeed, the biodegradability of the vegetable oils further increases the potential of AESO-based materials in this application field.

Hydrolytic degradation involves the cleavage of chemical bonds within the polymer through reaction with water. This process is influenced by factors such as the presence of hydrophobic groups and local pH variations, as well the molecular weight and architecture of the polymer [21]. Enzymatic degradation occurs when enzymes in the biological environment break down polymers by targeting specific bonds within their structure [22]. Oxidative degradation, typically linked to macrophage activity, involves peroxide radicals attacking covalent bonds in the polymer, aiding biomaterial clearance but potentially causing tissue damage if excessive [19]. Biodegradable materials offer temporary structural support during tissue regeneration, with customizable degradation rates for controlled bioactive molecule release, enhancing regeneration while minimizing side effects [17, 19, 23].

This study evaluated the biodegradability of two AESO formulations: pure AESO and AESO diluted with SO for suitable printability. Hydrolytic, enzymatic, and oxidative assays were performed to evaluate the *in vitro* biodegradation of the two formulations, as well as *in vitro* tests of cytocompatibility.

## 2. Experimental

### 2.1. Materials and Reagents

AESO (412333), SO (S7381), BAPO photoinitiator (phenyl bis(2,4,6-trimethylbenzoyl)phosphine oxide (511447)), KO_2_ (potassium dioxide (278904)), lipase from *Candida rugosa* (62302), PBS (phosphate-buffered saline) (P4417), NaN_3_ (sodium azide) (S2002), MTT (thiazolyl blue tetrazolium bromide) (M5655), and glutaraldehyde (G5882) were purchased from Sigma-Aldrich, Milan, Italy. Gel (G008 Eco & Ipl) was purchased from Fiab SpA, Florence, Italy. Ethanol, isopropanol, HCl (hydrochloric acid), and acetone were purchased from Carlo Erba, Milan, Italy. DMEM (Dulbecco's modified Eagle medium) and FBS (fetal bovine serum) were purchased from Thermo Fisher Scientific–Gibco, Milan, Italy. Penicillin/streptomycin solution and L-glutamine were purchased from Euroclone, Milan, Italy. A LIVE/DEAD Cell Imaging Kit (488/570) (R37601) was purchased from Thermo Fisher Scientific–Invitrogen, Milan, Italy.

### 2.2. Sample Preparation

Two types of samples were prepared for the experiments: AESO and AESO/SO (77.8/22.2% *w*/*w*); the AESO/SO ratio was decided according to the results obtained from the viscosity tests, described below. After the optimization of the concentration of the photoiniator BAPO (as described in Table S1 of Supporting Information (S1)) into AESO and AESO/SO formulations, BAPO was used at the final concentration of 1% *w*/*w* for both of them. The use of different solvents to favor the solubilization of BAPO into AESO and AESO/SO solutions was explored, also evaluating their effects on cell viability, as shown in Paragraph S2; Figures S1, S2, and S3; and Table S2 of Supporting Information. Since these preliminary results, it was decided to prepare the BAPO with no solvent, performing an overnight stirring incubation; then, the resulting preparations were allowed to rest for at least 48 h before use.

Different stainless steel and silicone molds were used to create disc-shaped or cylindrical-shaped specimens. The specific dimensions of the samples used in each test will be mentioned in the respective paragraphs.

For light curing, a light-emitting diode (LED) polymerization system (Aibecy) with a light intensity of 2200 mW/cm^2^ was employed. After a preliminary study regarding the irradiation time (as described in Table S1 of Supporting Information), the following curing conditions were applied: the samples were irradiated for 20 min, after which the upper surfaces were covered with a gel to prevent oxygen inhibition on the curing process. Subsequently, the samples were irradiated for an additional 10 min. After removing the gel layer, the samples were rinsed with dH_2_O and 100% ethanol for 5 min each. Finally, the samples were left to dry for at least 1 day before further use.

### 2.3. Methods

#### 2.3.1. Viscosity Test

To achieve a material that may be suitable for 3D printing via SLA/DLP, a viscosity test was conducted at room temperature (25°C) using a frequency sweep ranging from 0.1 to 100 Hz. As expected, the viscosity values of AESO alone exceeded the upper threshold of the viscosity parameters of most of the SLA/DLP 3D printers. As a result, AESO was mixed with SO using a planetary mixer (Thinky ARE 250) at 2000 rpm for 10 min. Subsequently, a defoaming cycle was conducted at 1500 rpm for 3 min. The test results revealed that a weight ratio of AESO : SO of 77.8 : 22.2% *w*/*w* provided an appropriate viscosity level for SLA/DLP printing (data not shown).

#### 2.3.2. Fourier-Transform Infrared (FTIR) Spectroscopy Analysis

To verify the successful polymerization, two samples of AESO and two samples of AESO/SO were prepared with the following dimensions: mass 35 mg; diameter 6 mm, and thickness 2.5 mm. Uncured and cured samples were analyzed using a Bruker ALPHA II compact FTIR spectrometer, equipped with an attenuated total reflection (ATR) module (Eco-ATR). All IR spectra were acquired in the wavelength range from 4000 to 650 cm^−1^ and, for each spectrum 54 scans, at a resolution of 2 cm^−1^ as averaged. The background was acquired before the measurements and then subtracted from each sample spectrum. The spectra were registered and preprocessed using the commercial OPUS 8.5 SP1 software, dedicated to the analysis of IR spectral data. Data were then exported and further analyzed with the programming software R. Spectra were normalized after linear baseline subtraction [24].

#### 2.3.3. Degradation Tests

For the degradation experiments in PBS, the sample dimensions were mass 150 mg, diameter 10 mm, and thickness 2.5 mm. For the enzymatic and oxidative degradation experiments, the sample dimensions were mass 100 mg, diameter 17 mm, and thickness 0.3 mm. All the samples have been sterilized with a UV lamp (254 nm) for 20 min. For all the degradation tests, the incubation was performed in the Labnet 311DS incubator (Spectra Service, 249 David Parkway Ontario, New York 14519), in which temperature accuracy is ±0.2°C and it is provided with a microprocessor as temperature controller. All tests were conducted in triplicate.

##### 2.3.3.1. Hydrolytic Degradation Test

After being weighed and sterilized, these samples were immersed in PBS (0.01 M, pH 7.4) in a ratio of 1 g : 10 mL [21]. The samples were left to degrade in the oven for 30 and 60 days at 37°C (to simulate a physiological condition) and 70°C (to simulate accelerated degradation) [25].

##### 2.3.3.2. Oxidative Degradation Test

After being weighed and sterilized, the samples were immersed in a 0.2 M KO_2_-based solution in PBS (0.15 M, pH 12) [26] and stirred at 70 rpm at 37°C for 60 days. The discs immersed in PBS (0.15 M, pH 12) were used as a control. The solutions were changed every 48/72 h, rinsing the discs from any degradation products with dH_2_O. To accurately assess the specimen's mass in all the conditions, they were rinsed with dH_2_O and left to dry in the oven at 30°C until a constant weight was obtained.

##### 2.3.3.3. Enzymatic Degradation Test

After being weighed and sterilized, the samples were dipped in 3 mL of PBS (0.1 M, pH 7.2) containing 0.25 mg/mL of lipase from *Candida rugosa*; 0.05% *w*/*w* sodium azide (NaN_3_) was added to check microbial activity and stirred at 70 rpm at 37°C for 60 days [27, 28]. 0.1 M PBS pH 7.2 solution was used as control. The solutions were changed every 48/72 h, rinsing the discs from any degradation products with dH_2_O. To accurately assess the specimen's mass in all the conditions, they were rinsed with dH_2_O and left to dry in the oven at 30°C until a constant weight was obtained.

After degradation with lipase, only the AESO/SO samples were subsequently immersed in a KO_2_-based solution (0.2 M) in PBS (0.15 M, pH 12) [26] for another 16 days.

#### 2.3.4. Cell–Material Interaction

##### 2.3.4.1. Cell Culture

3T3-Swiss mouse fibroblasts (kindly provided by Prof. Giovan Battista Pani, UCSC) were cultured at 37°C in a humidified atmosphere with 5% CO_2_, in DMEM high glucose, supplemented with 10% FBS, 100 units/mL penicillin, 100 *μ*g/mL streptomycin, and 2 mmol/L L-glutamine.

##### 2.3.4.2. Cytotoxicity Evaluation: Indirect Modality—MTT Assay

To evaluate the indirect cytotoxic effects of the materials, four samples of AESO and four samples of AESO/SO were prepared with the following dimensions: mass 35 mg, diameter 6 mm, and thickness 1 mm. After the synthesis, all discs were irradiated for 30 min with a UV lamp at 254 nm for the sterilization [29].

3T3-Swiss cells were seeded at a concentration of 2 × 10^4^ cells/well in DMEM, using a 24-well tissue culture plate, and cultured for 24 h until a subconfluent monolayer was formed. The disc-shaped specimens were placed into individual wells using a transwell insert, while cells without any material were used as controls. After 24 h of incubation, cell viability was evaluated by the MTT test, according to a previously described protocol [30]. Briefly, a solution of MTT in PBS (5 mg/mL) (100 *μ*L) was added to the medium (900 *μ*L) and, after an incubation of 4 h at 37°C, the produced intracellular formazan crystals were solubilized with a solution of HCl in isopropanol (4 × 10^−2^ M, 0.5 mL). The absorbance (ABS) of the solutions in each well was determined using an automatic microplate photometer (ELx800; BioTek, Bad Friedrichshall, Germany) at a wavelength of 570 nm. Each experiment was performed in quadruplicate and repeated three times, and the cytotoxicity was calculated according to the following equation [30]:

 $$\begin{array}{c} \%\text{cell mortality}=\left(\frac{{\text{ABS}}_{\text{control}}-{\text{ABS}}_{\text{sample}}}{{\text{ABS}}_{\text{control}}}\right)\times 100. \end{array}$$

Specimens were rated as slightly, moderately, or severely cytotoxic when the toxic effects relative to controls were < 30%, between 30% and 60%, or > 60%, respectively [31].

##### 2.3.4.3. Viability Evaluation: Direct Modality—Confocal Microscopy

To evaluate the viability of the cells grown on the materials, one disc of AESO and one of AESO/SO were prepared with the following dimensions: mass 100 mg, diameter 10 mm, and thickness 1.5 mm. After the synthesis, discs were irradiated for 30 min with a UV lamp at 254 nm for the sterilization [29].

3T3-Swiss cells were seeded on the produced discs at a concentration of 3 × 10^4^ cells/mL and cultured for 48 h; thus, cells were treated with the LIVE/DEAD Cell Imaging Kit (488/570) as indicated by the manufacturer. Cells grown on the samples have been observed at the Leica Confocal Microscope ctr 6500 TCS SP5. At least two images for each sample have been analyzed using Fiji/ImageJ, and mortality has been expressed in dead cells/total cells.

##### 2.3.4.4. Cell Adhesion

The samples utilized for this test had the following dimensions: mass 100 mg, diameter 10 mm, and thickness 1.5 mm. After the synthesis, all discs were irradiated for 30 min with a UV lamp at 254 nm for the sterilization [29].

To evaluate the interaction between 3T3-Swiss and the sterilized material discs, cells were seeded at a concentration of 1 × 10^4^ cells/well on AESO and AESO/SO samples, incubated at 37°C, 5% CO_2_, and maintained in culture for 2 days. The experiments were performed in duplicate.

Then, the specimens were prepared for scanning electron microscope (SEM) (Zeiss Supra 25) analysis as follows [32]: cells grown on samples were fixed by treating them with a 2% (*v*/*v*) glutaraldehyde solution in PBS (0.1 M, pH 7.4) for 5 min at 25°C. After washing with PBS, samples were dehydrated by exposing them for 15 min each to ethanol solutions with increasing concentration (from 60% to 100%) at room temperature. Then, samples were coated with a gold-particle layer using the Agar high-resolution sputter coater.

Reference samples without cells were prepared with the same procedure.

### 2.4. Statistical Analysis

Data are expressed as mean ± standard deviation (SD). Statistical analysis was performed using GraphPad software (GraphPad Prism Version 6.01 for Windows, GraphPad Software, La Jolla, California, United States). Means were compared using analysis of variance (ANOVA), followed, if necessary, by a multiple comparison of means using the Student–Newman–Keuls test.

Statistical significance is as follows: ⁣^∗^*p* < 0.05; ⁣^∗∗^*p* < 0.01; ⁣^∗∗∗^*p* < 0.001; ⁣^∗∗∗∗^*p* < 0.0001.

## 3. Results and Discussion

### 3.1. FTIR Analysis

**Figure 1:**
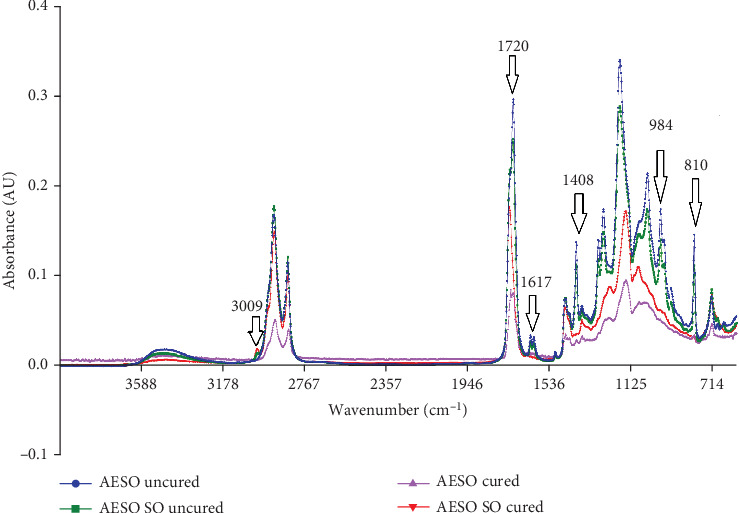
FTIR analysis. FTIR spectra of AESO and AESO/SO before and after UV curing.

Upon exposure of AESO and AESO/SO specimens to UV radiation in the presence of the photoinitiator BAPO, a swift free radical polymerization occurred. The FTIR spectra shown in Figure 1 illustrate the differences between AESO and AESO/SO before and after polymerization. The verification of polymerization was evident through a marked reduction in the intensity of acrylate functional group peaks at specific wavenumbers [33], including 984 and 810 cm^−1^, corresponding to out-of-plane deformation, 1406 cm^−1^ for in-plane CH_2_ deformation, 1617–1636 cm^−1^ associated with C=C stretching, and 1720 cm^−1^, which indicates the transformation of the acrylic functional group into a saturated ester group during polymerization. The significant reduction of these bands in cured specimens confirmed the conversion of double bonds. All signals related to liquid AESO/SO were less intense than those of pure AESO due to the dilution effect of SO. However, when considering the intensity of the peak at 3009 cm^−1^, the observed signal reduction is not due to the dilution of AESO but rather the epoxidation reaction; in fact, the signal at 3009 cm^−1^ indicates the presence of double bonds in SO. In AESO, these double bonds were converted in epoxy groups, and consequently, these signals are absent in AESO spectra. Since both SO and AESO are composed of triglycerides, the presence of signals at 1160 and 1744 cm^−1^ indicates the presence of ester groups C–O and C=O. The double bonds CH=CH, C=C, and =C–H are indicated by signals at wavenumbers 720, 1650, and 3010 cm^−1^, respectively [34, 35]. Furthermore, it is worth noting that the signals associated with pure AESO after polymerization are consistently lower compared to those of AESO/SO. This observation is expected, as it can be attributed to the presence of unmodified C=C double bonds in SO, which are inherently less reactive [36, 37].

### 3.2. Degradation Tests

**Figure 2:**
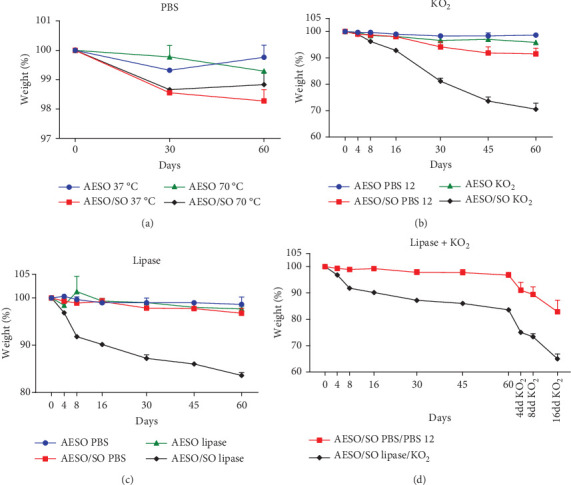
Degradation tests. The graphs show the effect of distinct types of degradation on AESO and AESO/SO in terms of weight loss percentage compared to the initial weight. Samples: Ctrl: AESO circle, AESO/SO square. Treatments: AESO triangle, AESO/SO rhombus. (a) Noncatalyzed hydrolytic degradation: samples have been dipped in a solution of PBS 0.01 M, pH 7.4, at 37°C and 70°C. (b) Oxidative degradation: samples have been dipped in a solution of PBS 0.15 M, pH 12, containing KO_2_ 0.2 M or not. (c) Enzymatic degradation: samples have been dipped in a solution of PBS 0.1 M, pH 7.2, containing lipase 0.25 mg/mL or not. (d) Coupled enzymatic and oxidative degradation: after the treatment described in (c), the same AESO/SO samples have been dipped in a solution of PBS 0.15 M, pH 12, containing KO_2_ 0.25 mg/mL or not, at the beginning and at the end of the incubation. The lines connecting the different time points are just meant as a guide to better follow the reduction of weight of the samples occurring during the four degradation assays. ⁣^∗∗^*p* < 0.01; ⁣^∗∗∗^*p* < 0.001; ⁣^∗∗∗∗^*p* < 0.0001. Unpaired Student's *t*-test analysis was used in the last picture.

Once implanted, biomaterials are usually target of different types of molecules, which may lead to its degradation, through hydrolytic, enzymatic, and oxidative processes [19]; indeed, numerous tests have been devised to replicate these degradation pathways *in vitro* [11, 26, 27, 38–41]. The *in vitro* degradation of AESO or similar compounds has shown to vary in a significant way, depending on the different study conditions [9, 11, 27, 40]. In our case, two formulations—AESO and AESO/SO—have been tested for hydrolytic, oxidative, and enzymatic degradation. The noncatalyzed hydrolytic degradation of AESO and AESO/SO samples has been conducted in a solution of PBS 0.1 M at 37°C (to simulate physiological conditions) and 70°C (to obtain an accelerated degradation) and is shown in Figure 2(a). The temperature-caused degradation is extremely low in all the conditions: indeed, the AESO/SO 37°C is the one with the highest degradation, with a weight loss of only 1.73% after 60 days of treatment. These results indicated that both materials are not sensible to noncatalyzed hydrolysis and the temperature does not influence the outcome. Even if the ester bonds residing at the fatty acid–glycerol and fatty acid–acrylic acid linkages may render the whole structure subjective to this noncatalyzed hydrolytic degradation [11, 38, 40, 42, 43], the hydrophobic nature [44], the low water absorption capacity [10, 45, 46], and the covalent network obtained via radical photopolymerization [47, 48] make these polymers quite resistant to a noncatalyzed hydrolytic degradation. Indeed, these data corroborate with another study conducted on a similar material: Mondal et al. observed only a 0.3% of weight loss after dipping AESO/nanoHA/PEGDA samples in distilled water for 28 days [11]. Moreover, Bragaglia et al. observed that the 3D-printed AESO/SO samples are sensible to NaOH-catalyzed hydrolytic degradation [9].

Upon the biomaterial implantation, neutrophils and activated macrophages initiate the inflammatory response and start to release, along with the enzymes, different types of reactive oxygen species (ROS) and reactive nitrogen species (RNS), which may induce the oxidative degradation of the implanted polymer [38, 39]. Both AESO and, in particular, AESO/SO seem to be susceptible to superoxide anions (•O_2−_) produced by potassium dioxide (KO_2_) (Figure 2(b)). Indeed, the AESO/SO samples show a weight loss of 29.45% after 60 days of KO_2_ treatment, while the AESO ones lost only 4.12% of their initial weight; moreover, the degradation observed in both formulations in the presence of KO_2_ is statistically significant (*p* < 0.0001 for the former and *p* < 0.01 for the latter). The superoxide anions play a role in these polymers' degradation, and this process can be chemically explained by the nucleophilic attack of •O_2−_ to the carbonyl group of the ester bonds [49]. Part of the AESO/SO degradation is caused by the alkaline environment, which does simulate an accelerated *in vitro* hydrolytic degradation [9, 40, 41]; indeed, the AESO/SO samples lost around 8.47% of their weight in the control condition (*p* < 0.0001), while AESO samples show 1.34% of degradation (*p* > 0.05) in 60 days. Besides, while the AESO's weight loss does not differ in a significant manner after 60 days between the treatment and control conditions (*p* > 0.05), AESO/SO shows a 20.98% of difference of weight reduction in the same conditions (*p* < 0.0001). The reason why AESO/SO polymer is more sensible compared to its neat form that resides in SO, which only partially participates in the photopolymerization process, is because of the low reactivity of the fatty acid double bounds [36, 37]—as it was already demonstrated by the FTIR analysis (Figure 1)—leading to a lower crosslink density. This leads to think that the diluent is most degraded in the AESO/SO blend, even if also the AESO has to be sensible to the superoxide anions, as the SO weight percentage in the AESO/SO blend accounts for 22.2% and AESO/SO samples lose 29.45% of their weight after 60 days.

Activated macrophages and neutrophils release diverse types of hydrolytic enzymes—such as proteases, esterases, and lipases—which take part in the degradation of the implanted material [38, 39]. Upon 60 days of treatment, the AESO/SO lipase-induced weight loss settles at 15.5% (*p* < 0.0001), while the AESO's one settles at 2.72% (*p* < 0.001) (Figure 2(c)). Also, AESO/SO showed a significative reduction (*p* < 0.001) even in a physiological buffer, while AESO does not exhibit a significant decline in this condition (*p* > 0.05). Moreover, the difference of weight reduction of AESO/SO between the treatment and the control conditions after 60 days is significant (*p* < 0.0001), contrary to the one of AESO (*p* > 0.05). As for the oxidative degradation, the higher sensibility of AESO/SO is an effect of the presence of SO, which allows for a lower crosslink density leading to higher diffusion of the enzyme [21, 27]. Nevertheless, the lipase-induced degradation is less effective compared to the KO_2_-induced one: this result can be explained by the more aggressive alkaline environment of the oxidative degradation test and by the fact that lipase from *Candida rugosa* is a nonspecific enzyme and the substrate specificity nature of the enzymes affects the degradation rate [21, 50]. In either case, this AESO/SO blend is more sensible to the lipase action compared to other copolymers of AESO evaluated in previous studies [11, 27].

Since the hydrolytic, enzymatic, and oxidative degradations occur simultaneously in physiological conditions, the lipase-predigested AESO/SO discs have been treated with KO_2_ (Figure 2(d))—the AESO ones have not been analyzed as they did not show a remarkable lipase degradation. Looking at the curve slope, it is evident how the weight loss rate increases upon the change of treatment; indeed, the AESO/SO samples lost 18.62% of weight in 16 days of KO_2_ (*p* < 0.001) and 35.02% compared to the initial weight (*p* < 0.0001), while its control lost 19.89% (*p* < 0.01) and 17.12% (*p* < 0.0001), respectively. This is an interesting finding as it unequivocally demonstrates that degradation affects both AESO and SO and not just SO. Therefore, under conditions closer to physiological ones, the material may undergo complete degradation, making this composition a suitable option as biodegradable implantable biomaterial.

### 3.3. Cell–Material Interaction

#### 3.3.1. Cytotoxic Evaluation in Indirect and Direct Assays

The viability of cells cultured on the discs was assessed using both indirect and direct methods at 24 and 48 h of cell growth, respectively.

**Figure 3:**
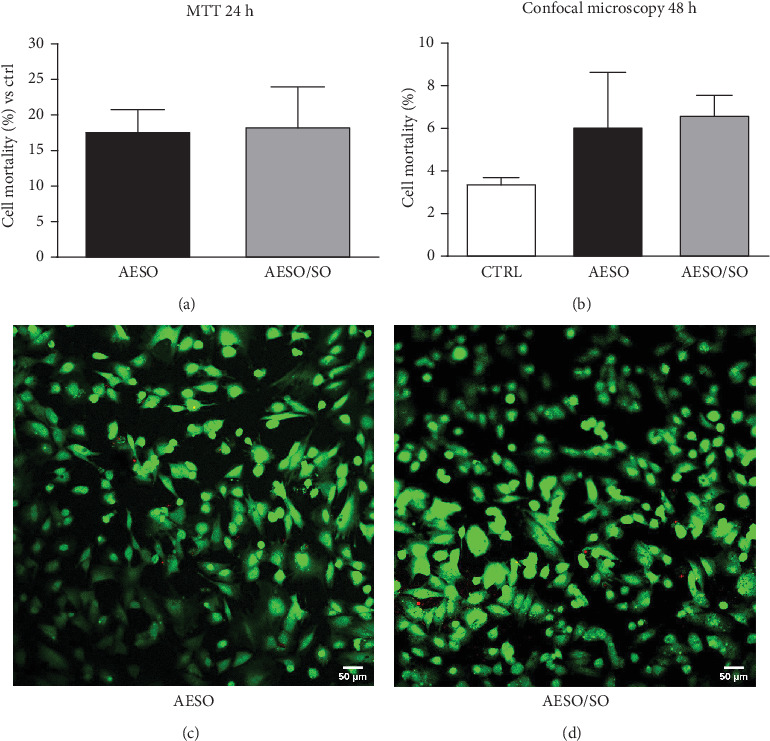
Cell viability. (a) MTT assay after 24 h of cell culture, indicating the percentage of cell mortality compared to the control (no material) for cells grown on AESO and AESO/SO samples. (b) LIVE/DEAD assay confocal microscopy images after 48 h of cell culture showing the relative cell mortality for cells grown on the glass cover slide control, AESO and AESO/SO samples. The mortality is calculated as the ratio of dead cells to total cells. (c, d) Confocal microscopy images displaying live cells (green) and dead cells (red) for cells grown on (c) AESO and (d) AESO/SO samples.

The two methods for toxicity evaluation provide complementary and intriguing information. Toxicity assessed through indirect modality has allowed us to determine that both materials do not release toxic substances. In fact, the calculated toxicity was 17.73% in the presence of AESO and 18.40% in the presence of AESO/SO samples (Figure 3(a)). These cytotoxic values can be considered slight, according to the Sletten classification [31]. No statistical differences are present between the two materials.

The 24-h contact time between material eluates and cells was chosen in accordance with the literature data, because, as well known, the release of nonpolymerized molecules from photopolymerized materials occurs during the initial 24 h [51].

Cytotoxicity assessed through the direct method allowed us to evaluate whether the material can support cell survival. The chosen 48-h period for this test was determined to study acute toxicity, providing sufficient time for cell adhesion and potential initiation of mitotic processes. Given the distinct nature of the information provided by the two tests, direct toxicity was not evaluated using the MTT test, which is a relative assay. Instead, we employed a test that enables the direct counting of viable and dead cells on the materials. The cells grown on AESO and AESO/SO samples displayed similar mortality rates of 6.09% and 6.64%, respectively, while the mortality of the control group (cells grown on a glass cover slide) is 3.43% (Figure 3(b)). Figures 3(c) and 3(d) show cells grown on AESO and AESO/SO samples, respectively. No statistical differences are present among the two materials and the control group. These results are concordant with the observed good proliferation capacity of 3T3-L1 fibroblasts cultured on AESO/SO specimen [9].

Due to the different endpoints evaluated, there is not an absolute agreement between the results obtained with the two systems. However, each method provides unique information and insights into the toxic effects of the materials and, when considered together, they reinforce the evidence of the low cytotoxicity of AESO and AESO/SO.

#### 3.3.2. Cell Adhesion

**Figure 4:**
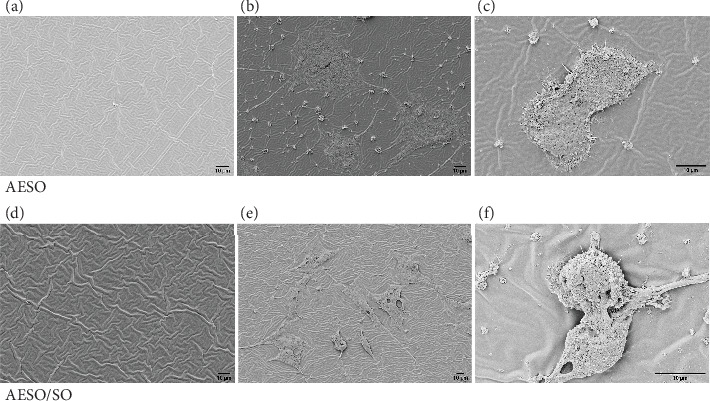
Cell adhesion. SEM images after 48 h of cell culture showing the adhesion of the 3T3 cells to (b, c) AESO and (e, f) AESO/SO samples. Reference SEM images show (a) AESO and (d) AESO/SO surface with no cells grown on them.

To evaluate the adhesion of the 3T3-Swiss cells to the AESO and AESO/SO discs after 48 h of cell culture, we observed samples at the SEM (Figure 4)—control samples maintained in the growth medium in the absence of cells have been used as negative controls (AESO, Figure 4(a); AESO/SO, Figure 4(d)).

Based on the SEM micrographs provided, 3T3-Swiss fibroblasts not only adhere to the discs made of AESO (Figure 4(b)) and AESO/SO (Figure 4(e)), but they were also observed to undergo mitosis, as depicted in Figure 4(c) (AESO) and Figure 4(f) (AESO/SO). This intriguing experimental finding suggests that the material exhibits favorable cytocompatibility properties, which was further supported by the MTT and confocal microscopy results.

## 4. Conclusions

The study of material biodegradability is of utmost importance, especially in the realm of biomedical applications. Our research has highlighted how AESO, when used as a biomaterial, undergoes a distinctive degradation process that combines both enzymatic and oxidative degradation. This phenomenon results from a complex interplay of factors, including the chemical composition and physical characteristics of the polymer, the unique tissue environment in which it is implanted, and the duration of implantation.

Understanding and being able to manipulate these intricate reactions are of paramount importance in designing biodegradable biomaterials with customized degradation rates and elimination profiles. This profound comprehension opens the door to the development of biomaterials capable of providing temporary support, promoting tissue regeneration and mitigating adverse effects throughout the healing process.

Our work has unequivocally demonstrated that both pure and diluted AESO can degrade under conditions similar to those found in biological systems. The importance of studying the degradation processes of biomaterials is fundamental for designing safe, effective, and customizable materials, thereby improving the effectiveness of biomedical therapies and the quality of life for patients.

The significance of this study lies in the detailed analysis that allowed us to identify the specific dynamics regulating the degradation of AESO. Our research extends beyond superficial observations, exploring the chemical and physical mechanisms involved and providing a foundation for developing new generations of biomaterials. These materials need also to be capable of degrading in a controlled and predictable manner, adapting to the specific needs of the patient and the biological environment in which they are used.

In conclusion, our research contributes to the development of advanced biomimetic materials with potential applications in biomedicine. By enhancing the understanding of AESO degradation, this study is aimed at improving the safety and efficacy of medical treatments, offering innovative and customizable solutions to address clinical challenges.

## Data Availability

Data are available on request.

## Supporting Information

Supporting Information Additional supporting information can be found online in the Supporting Information section. Table S1. Thickness and irradiation time tests of AESO with different BAPO concentrations. Figure S1. Staining due to the reaction of the cells seeded on the three discs prepared with Solutions 1 (AESO : acetone), 2 (AESO : diethyl ether), and 3 (AESO : no solvent with MTT after 1 week). The disc of Solution 3 appears more purple, that is, with more viable cells. Figure S2. Cytotoxic effect with MTT test of AESO discs prepared with Solutions 1 (AESO : acetone), 2 (AESO : diethyl ether), and 3 (AESO : no solvent determined in indirect modality). Table S2. Cytotoxic effect with MTT test of AESO discs prepared with Solutions 1 (AESO : acetone), 2 (AESO : diethyl ether), and 3 (AESO : no solvent determined in eluate modality). Figure S3. Staining due to the reaction of the cells (seeded on the discs) with MTT after 1 week (A) and after 2 weeks (B). The discs present in picture B appear more purple, that is, with more viable cells.

## References

[1] Odabasoglu F., Halici Z., Cakir A. (2008). Beneficial effects of vegetable oils (corn, olive and sunflower oils) and *α*-tocopherol on anti-inflammatory and gastrointestinal profiles of indomethacin in rats. *European Journal of Pharmacology*.

[2] Ribeiro A. R., Silva S. S., Reis R. L. (2022). Challenges and opportunities on vegetable oils derived systems for biomedical applications. *Biomaterials Advances*.

[3] Nurchi C., Buonvino S., Arciero I., Melino S. (2023). Sustainable vegetable oil-based biomaterials: synthesis and biomedical applications. *International Journal of Molecular Sciences*.

[4] Rahimi M., Pirouzfar V., Sakhaeinia H. (2024). Acrylated epoxidized soybean oil/polyvinylidene fluoride nanocomposites: demonstrating the potential role of plant oils in biomedical applications. *Journal of Polymers and the Environment*.

[5] Mendes-Felipe C., Costa P., Roppolo I., Sangermano M., Lanceros-Mendez S. (2022). Bio-based piezo- and thermoresistive photocurable sensing materials from acrylated epoxidized soybean oil. *Macromolecular Materials and Engineering*.

[6] Haghpanah Z., Mondal D., Momenbeitollahi N. (2024). In vitro evaluation of bone cell response to novel 3D-printable nanocomposite biomaterials for bone reconstruction. *Journal of Biomedical Materials Research Part A*.

[7] Biermann U., Bornscheuer U., Meier M. A. R., Metzger J. O., Schäfer H. J. (2011). Oils and fats as renewable raw materials in chemistry. *Angewandte Chemie International Edition*.

[8] Miao S., Zhu W., Castro N. J. (2016). 4D printing smart biomedical scaffolds with novel soybean oil epoxidized acrylate. *Scientific Reports*.

[9] Bragaglia M., Sciarretta F., Filetici P. (2024). Soybean oil-based 3D printed mesh designed for guided bone regeneration (GBR) in oral surgery. *Macromolecular Bioscience*.

[10] Mondal D., Srinivasan A., Comeau P., Toh Y. C., Willett T. L. (2021). Acrylated epoxidized soybean oil/hydroxyapatite-based nanocomposite scaffolds prepared by additive manufacturing for bone tissue engineering. *Materials Science and Engineering: C*.

[11] Mondal D., Haghpanah Z., Huxman C. J. (2021). MSLA-based 3D printing of acrylated epoxidized soybean oil - nano-hydroxyapatite composites for bone repair. *Materials Science and Engineering: C*.

[12] Kasetaite S., De la Flor S., Serra A., Ostrauskaite J. (2018). Effect of selected thiols on cross-linking of acrylated epoxidized soybean oil and properties of resulting polymers. *Polymers*.

[13] Li G., Li Z., Min Y., Chen S., Han R., Zhao Z. (2023). 3D-printed piezoelectric scaffolds with shape memory polymer for bone regeneration. *Small*.

[14] Fernandes T. A., Costa I. F. M., Jorge P. (2021). Silver(I) coordination polymers immobilized into biopolymer films for antimicrobial applications. *ACS Applied Materials & Interfaces*.

[15] Fernandes T. A., Costa I. F. M., Jorge P. (2022). Hybrid silver(I)-doped soybean oil and potato starch biopolymer films to combat bacterial biofilms. *ACS Applied Materials & Interfaces*.

[16] Drelich J. W., Sikora-Jasinska M., Mostaed E. (2020). Biodegradable materials for medical applications II. *JOM*.

[17] Modrák M., Trebuňová M., Balogová A. F., Hudák R., Živčák J. (2023). Biodegradable materials for tissue engineering: development, classification and current applications. *Journal of Functional Biomaterials*.

[18] Tajvar S., Hadjizadeh A., Samandari S. S. (2023). Scaffold degradation in bone tissue engineering: an overview. *International Biodeterioration & Biodegradation*.

[19] Li C., Guo C., Fitzpatrick V. (2020). Design of biodegradable, implantable devices towards clinical translation. *Nature Reviews Materials*.

[20] Göpferich A. (1996). Mechanisms of polymer degradation and erosion. *Biomaterials*.

[21] Brannigan R. P., Dove A. P. (2016). Synthesis, properties and biomedical applications of hydrolytically degradable materials based on aliphatic polyesters and polycarbonates. *Biomaterials Science*.

[22] Liu M., Zhang T., Long L., Zhang R., Ding S. (2019). Efficient enzymatic degradation of poly (ɛ-caprolactone) by an engineered bifunctional lipase-cutinase. *Polymer Degradation and Stability*.

[23] Mansour A., Romani M., Acharya A. B., Rahman B., Verron E., Badran Z. (2023). Drug delivery systems in regenerative medicine: an updated review. *Pharmaceutics*.

[24] Di Santo R., Vaccaro M., Romanò S. (2022). Machine learning-assisted FTIR analysis of circulating extracellular vesicles for cancer liquid biopsy. *Journal of Personalized Medicine*.

[25] (2010). ISO 10993-13:2010 Biological evaluation of medical devices Part 13: Identification and quantification of degradation products from polymeric medical devices.

[26] Beck-Broichsitter M. (2020). Comparative *in vitro* degradation of surface-eroding poly(alkylene carbonate)s. *Polymer Degradation and Stability*.

[27] Kim H.-M., Kim H.-R., Hou C. T., Kim B. S. (2010). Biodegradable photo-crosslinked thin polymer networks based on vegetable oil hydroxy fatty acids. *Journal of the American Oil Chemists’ Society*.

[28] Kim H.-M., Kim H.-R., Kim B. S. (2010). Soybean oil-based photo-crosslinked polymer networks. *Journal of Polymers and the Environment*.

[29] Huebsch N., Gilbert M., Healy K. E. (2005). Analysis of sterilization protocols for peptide-modified hydrogels. *Journal of Biomedical Materials Research Part B: Applied Biomaterials*.

[30] Marigo L., Spagnuolo G., Malara F. (2015). Relation between conversion degree and cytotoxicity of a flowable bulk-fill and three conventional flowable resin-composites. *European Review for Medical and Pharmacological Sciences*.

[31] Sletten G. B. G., Dahl J. E. (1999). Cytotoxic effects of extracts of compomers. *Acta Odontologica Scandinavica*.

[32] Chronopoulou L., Cacciotti I., Amalfitano A. (2021). Biosynthesis of innovative calcium phosphate/hydrogel composites: physicochemical and biological characterisation. *Nanotechnology*.

[33] Barkane A., Platnieks O., Jurinovs M. (2021). UV-light curing of 3D printing inks from vegetable oils for stereolithography. *Polymers*.

[34] Saha P., Kim B. S. (2019). Preparation, characterization, and antioxidant activity of *β*-carotene impregnated polyurethane based on epoxidized soybean oil and malic acid. *Journal of Polymers and the Environment*.

[35] Bach Q.-V., Vu C. M., Vu H. T., Hoang T., Dieu T. V., Nguyen D. D. (2020). Epoxidized soybean oil grafted with CTBN as a novel toughener for improving the fracture toughness and mechanical properties of epoxy resin. *Polymer Journal*.

[36] Saithai P., Lecomte J., Dubreucq E., Tanrattanakul V. (2013). Effects of different epoxidation methods of soybean oil on the characteristics of acrylated epoxidized soybean oil-co-poly(methyl methacrylate) copolymer. *Express Polymer Letters*.

[37] Zhao Y. H., Hupin S., Lecamp L. (2017). Thiol–ene chemistry of vegetable oils and their derivatives under UV and air: a model study by using infrared spectroscopy and mass spectrometry. *RSC Advances*.

[38] Brugmans M. C. P., Sӧntjens S. H. M., Cox M. A. J. (2015). Hydrolytic and oxidative degradation of electrospun supramolecular biomaterials: in vitro degradation pathways. *Acta Biomaterialia*.

[39] Bat E., van Kooten T. G., Feijen J., Grijpma D. W. (2009). Macrophage-mediated erosion of gamma irradiated poly(trimethylene carbonate) films. *Biomaterials*.

[40] Yang G., Rohde B. J., Robertson M. L. (2013). Hydrolytic degradation and thermal properties of epoxy resins derived from soybean oil. *Green Materials*.

[41] Shen M., Almallahi R., Rizvi Z., Gonzalez-Martinez E., Yang G., Robertson M. L. (2019). Accelerated hydrolytic degradation of ester-containing biobased epoxy resins. *Polymer Chemistry*.

[42] Lyu S., Untereker D. (2009). Degradability of polymers for implantable biomedical devices. *International Journal of Molecular Sciences*.

[43] Zhang C., Garrison T. F., Madbouly S. A., Kessler M. R. (2017). Recent advances in vegetable oil-based polymers and their composites. *Progress in Polymer Science*.

[44] Omer R. A., Hughes A., Hama J. R., Wang W., Tai H. (2015). Hydrogels from dextran and soybean oil by UV photo-polymerization. *Journal of Applied Polymer Science*.

[45] Ito S., Hashimoto M., Wadgaonkar B. (2005). Effects of resin hydrophilicity on water sorption and changes in modulus of elasticity. *Biomaterials*.

[46] Dhanpal P., Yiu C. K. Y., King N. M., Tay F. R., Hiraishi N. (2009). Effect of temperature on water sorption and solubility of dental adhesive resins. *Journal of Dentistry*.

[47] van Bochove B., Grijpma D. W. (2019). Photo-crosslinked synthetic biodegradable polymer networks for biomedical applications. *Journal of Biomaterials Science, Polymer Edition*.

[48] Shogren R. L., Petrovic Z., Liu Z., Erhan S. Z. (2004). Biodegradation behavior of some vegetable oil-based polymers. *Journal of Polymers and the Environment*.

[49] Lee K. H., Won C. Y., Chu C. C., Gitsov I. (1999). Hydrolysis of biodegradable polymers by superoxide ions. *Journal of Polymer Science Part A: Polymer Chemistry*.

[50] Domínguez de María P., Sánchez-Montero J. M., Sinisterra J. V., Alcántara A. R. (2006). Understanding *Candida rugosa* lipases: an overview. *Biotechnology Advances*.

[51] Ferracane J. L., Condon J. R. (1990). Rate of elution of leachable components from composite. *Dental Materials*.

