# Supplementary material for: Biodegradation Study of Biomaterials Composed of Acrylated Epoxidized Soybean Oil: An In Vitro Study
Source: Biomed Res Int. 2024 Nov 22;2024:7100988. doi: 10.1155/bmri/7100988 (PMC11608301; doi:10.1155/bmri/7100988)
Supplement: Supporting Information — Additional supporting information can be found online in the Supporting Information section. Table S1. Thickness and irradiation time tests of AESO with different BAPO concentrations. Figure S1. Staining due to the reaction of the cells seeded on the three discs prepared with Solutions 1 (AESO : acetone), 2 (AESO : diethyl ether), and 3 (AESO : no solvent with MTT after 1 week). The disc of Solution 3 appears more purple, that is, with more viable cells. Figure S2. Cytotoxic effect with MTT test of AESO discs prepared with Solutions 1 (AESO : acetone), 2 (AESO : diethyl ether), and 3 (AESO : no solvent determined in indirect modality). Table S2. Cytotoxic effect with MTT test of AESO discs prepared with Solutions 1 (AESO : acetone), 2 (AESO : diethyl ether), and 3 (AESO : no solvent determined in eluate modality). Figure S3. Staining due to the reaction of the cells (seeded on the discs) with MTT after 1 week (A) and after 2 weeks (B). The discs present in picture B appear more purple, that is, with more viable cells. [file 7100988.f1.docx]

**Supplementary Materials**

**S1. Identification of the best curing parameters and sample dimensions**

To obtain a better resin composition, a plethora of mixtures was prepared utilizing different stoichiometric ratios between AESO and BAPO, as well different irradiation times and thickness of the samples. All the utilized conditions were reported in Table S1. Different concentrations (1, 0.50 or 0.10 % w/w) of the photoinitiator Phenyl bis(2,4,6-trimethylbenzoyl) phosphine oxide (BAPO), were used in the compositions.

|  | **BAPO Concentration (% w/w)** | | | | | | | | | | | |
| --- | --- | --- | --- | --- | --- | --- | --- | --- | --- | --- | --- | --- |
|  | **1.00 %** | | | | **0.5 %** | | | | **0.1 %** | | | |
| **Thickness (mm)** | 0.1 | 1 | 2 | 4 | 0.1 | 1 | 2 | 4 | 0.1 | 1 | 2 | 4 |
| **Irradiation time (min)** | 1 | 10 | 20 | 30 | 1 | 10 | 20 | 30 | 1 | 10 | 20 | 30 |

TABLE S1 – Thickness and irradiation time tests of AESO samples with different BAPO concentrations.

The qualitative results obtained with the 1 % BAPO concentration turned out to be always the best (data not shown). Therefore, all subsequent experiments were carried out using the photoinitiator at the final concentration of 1 % and applying an irradiation time equal to 30 minutes, while the sample thickness was modified according to the experimental needs.

**S2. Identification of the best BAPO solubilization condition**

To optimize the solubilization of BAPO, the following conditions were tested:

1. Solution (1): AESO:acetone in a w:v ratio of 1:1.
2. Solution (2): AESO:diethyl ether (T_eb_ lower than acetone) ratio of 1:1 w:v.
3. Solution (3): AESO:no solvent.

To remove the two solvents, the mixtures were stirred for 6 hours and then left to rest for 48 hours. After polymerization, the samples were washed two times with 100 % ethanol for 5 minutes. Thus, resins were exposed to UV rays (wavelength 254 nm) for 2h for the sterilization, to simulate the subsequent cytotoxicity assays.

A small amount of both solvents was found within the solutions (1) and (2); in particular, the residual concentration of acetone in Solution (1) was 1.52 % and that of diethyl ether was 0.84 % in Solution (2).

**S3. Cytotoxic evaluation**

**S3.1 Direct tests – Qualitative determination**

The cellular toxicity induced by the three different BAPOS’s solubilization conditions was evaluated. The dimensions of the discs were: diameter 10 mm, thickness 2 mm.

3T3-Swiss cells were seeded on the discs (2 x 10^4^ cells/disc) in a 24-well plate and the MTT assay was performed after one week. The obtained results (Figure S1) showed a greater proliferation of cells on the disc obtained by adding BAPO directly to AESO (Solution (3).


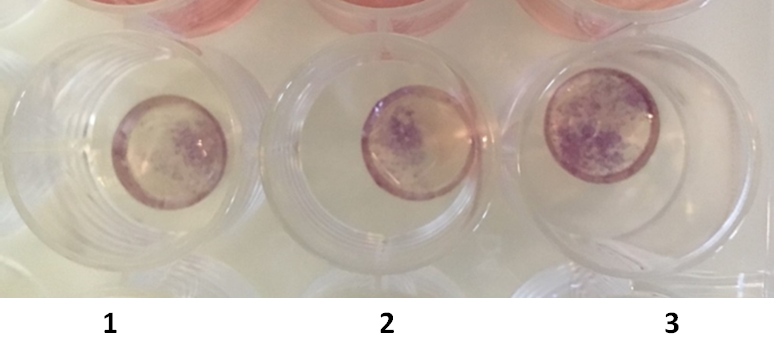


FIGURE S1 – Staining due to the reaction of the cells with MTT after 1 week upon seeding on the three discs prepared with Solution (1) (AESO:acetone), (2) (AESO:diethyl ether) and (3) AESO:no solvent. The disc of Solution (3) appears purpler, i.e. with more viable cells.

**S3.2 Indirect tests - Quantitative determination**

3T3-Swiss cells were seeded in 24-well plates (2 x 10^4^ cells/well) and grown for 24h. At the end of this period, the discs were added to the cell culture and left for other 24h; after that, the MTT assay was performed.

Obtained results, reported in Figure S2, are coherent with the qualitative results obtained in a direct way. In fact, the mortality induced by the discs of Solution (1) is moderately higher (19.61 %) compared to discs in Solution (2) and (3), which showed a very light cytotoxic effect, with a mortality of 6.75 % and 1.55 %, respectively.

FIGURE S2 – Cytotoxic effect of AESO discs prepared with Solution (1) (AESO:acetone), (2) (AESO:diethyl ether) and (3) (AESO:no solvent) determined in indirect modality via the MTT test.

**S3.3 Eluate tests – Quantitative determination**

The discs prepared with Solution (1), (2) and (3) were left to elute for 24 h in cell-free culture medium at 37 ° C with a surface/volume ratio of 0.5 cm^2^/mL. The total surface of the prepared discs was 2.2 cm^2^, therefore a volume of 4.4 mL of medium was added to each disc.

3T3-Swiss cells were seeded in 24-well plates (2 x 10^4^ cells/well) and grown for 24h, thus the medium deriving from the eluate was added and, after another 24 h, the MTT assay was performed. Results are reported in Table S2. The test did not reveal any toxic effects.

|  | **AESO:Acetone Sol (1)** | **AESO:Diethyl Ether Sol (2)** | **AESO:No Solv Sol (3)** |
| --- | --- | --- | --- |
| **Mortality** | -0.06157 % ± 4.336 | -1.063 % ± 6.808 | + 2.503 % ± 4.044 |

TABLE S2 – Cytotoxic effect of AESO discs prepared with Solution (1) (AESO:acetone), (2) (AESO:diethyl ether) and (3) (AESO:no solvent) determined in eluate modality via the MTT test.

**S3.4 Proliferation assay**

Cell proliferation on AESO and AESO/SO samples was evaluated over a period of 7 and 15 days to demonstrate the materials’ capability to support cell growth for an extended duration, using the MTT test in a qualitative manner.

3T3-Swiss cells were seeded on the discs (2 x 10^4^ cells/disc) in 12-well plates and grown for 1 week (Figure S3A) and 2 weeks (Figure S3B). Obtained results showed the ability of the two formulations to allow cell proliferation for a long period of culture.


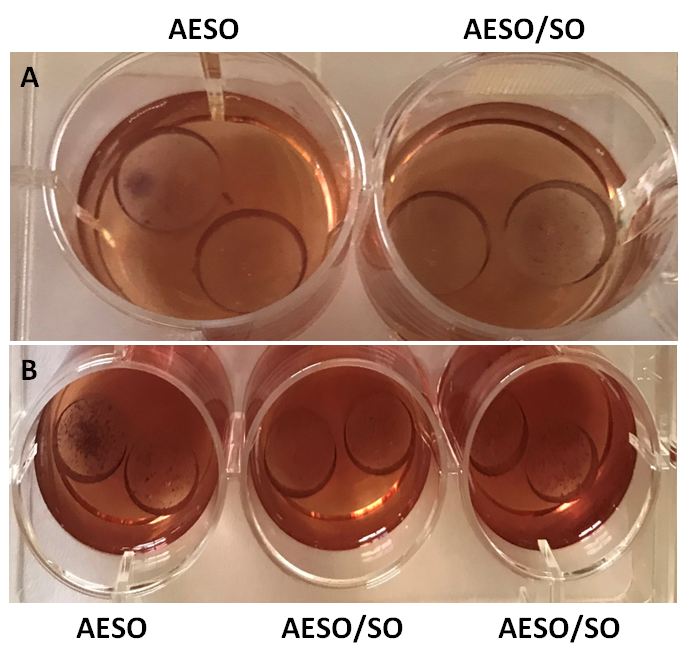


FIGURE S3 – Staining due to the reaction of the cells with MTT after 1 week (A) and after 2 weeks (B) upon seeding on AESO and AESO/SO samples. The discs present in the B picture appear purpler, i.e. with more viable cells.
